# MiR‐191‐5p inhibits lung adenocarcinoma by repressing SATB1 to inhibit Wnt pathway

**DOI:** 10.1002/mgg3.2111

**Published:** 2022-11-28

Zhou, L. Y., Zhang, F. W., Tong, J., Liu, F. “MiR‐191‐5p inhibits lung adenocarcinoma by repressing SATB1 to inhibit Wnt pathway”. *Molecular Genetics & Genomic Medicine*. 2020, 8(1), e1043. https://doi.org/10.1002/mgg3.1043

The above article, published online on 13 November 2019 in Wiley Online Library (https://onlinelibrary.wiley.com), has been retracted by agreement between the journal Editor‐in‐Chief Dr. Suzanne Hart and John Wiley & Sons. The retraction has been agreed due to concerns raised by a third party about image manipulation. The journal team has investigated and determined that the image manipulation undermines the reliability of the data presented and the article's conclusions.

